# Association between gastrointestinal diseases and osteoarthritis risk based on data from NHANES 2011–2018

**DOI:** 10.1371/journal.pone.0330064

**Published:** 2025-08-13

**Authors:** Mingzhu Lu, Yangkun Ding

**Affiliations:** Department of Pediatric Orthopaedics, Jinan Children’s Hospital, Jinan, China; University of Life Sciences in Lublin, POLAND

## Abstract

Osteoarthritis (OA) and gastrointestinal diseases are two significant public health problems. However, the association between the two is unclear. This study aimed to investigate the biological relationship between gastrointestinal diseases and OA using data derived from the National Health and Nutrition Examination Survey (NHANES) database. A total of 8,833 participants registered in the NHANES between 2011 and 2018 were enrolled; 5,044 participants were included in the study after excluding ineligible samples. Three models were constructed to investigate the correlation between gastrointestinal diseases and OA. Risk stratification analysis was conducted, and a receiver operating characteristic (ROC) curve was employed. Among 5,044 participants, even after stratification, gastrointestinal diseases significantly affected OA occurrence; this effect persisted despite adjusting for all covariates in model 3 (odds ratio = 2.01; 95% confidence interval = 1.23–3.3, *p* = 0.0069) and was confirmed by risk-stratification analysis. The ROC curve and the smooth curve showed that gastrointestinal diseases increased OA risk. Gastrointestinal diseases are significant risk factors for OA, which indicates a potential theoretical basis for OA prevention.

## Introduction

Osteoarthritis (OA) is a prevalent chronic joint disorder characterized by degradation of articular cartilage and alterations in the subchondral bone and synovium [1,2]. Approximately 500 million people (7% of the global population) suffer from OA, which is the leading cause of joint disability in older adults and some children and negatively affects mental health [1,3,4]. Several studies have reported a multifactorial etiology for OA, including aging, obesity, joint dysplasia, trauma, and physical activity, but the definite etiology remains unclear. Therefore, exploring and clarifying the causative factors for OA are beneficial for the clinical prevention and treatment of OA [3,4].

Gastrointestinal diseases encompass a wide range of benign and malignant conditions affecting the digestive tract, liver, biliary system, and pancreas. These diseases are typically characterized by abdominal pain, nausea, vomiting, diarrhea, and constipation, and may significantly impact an individual’s digestive and absorptive functions [5]. With lifestyle changes, the incidence of gastrointestinal diseases, including peptic ulcer disease, gastroesophageal reflux disease, and inflammatory bowel disease (IBD), is also increasing [6–8]. Gastrointestinal diseases are an important public health issue,because they not only increase the global disease and socioeconomic burden but also have a profound impact on the daily lives and mental health of patients.

Acute or chronic gastrointestinal symptoms, such as vomiting and diarrhea, may indicate an imbalance in the intestinal microbiota [9,10].Previous studies have mostly focused on the prevalence of single diseases, with only a few observational studies indicating that gastrointestinal diseases may indirectly contribute to the progression of OA via inflammatory and immune responses and the gut microbiota [11–13]. The gut microbiota produces essential compounds, including enzymes and short-chain fatty acids. However, harmful bacteria can produce pro-inflammatory substances (such as lipopolysaccharides) that enter the bloodstream via “leaky gut,” triggering systemic inflammation. This inflammation can impact joints, potentially leading to diseases such as OA, rheumatoid arthritis, and spondyloarthritis. The association between gastrointestinal diseases and OA is a vital research area, emphasizing the potential role of the gut microbiota in immune regulation and joint health [14–16].However, as only small-sample studies have been conducted,the reliability of the results may be questionable. Further large-scale studies to confirm the relationship between gastrointestinal diseases and OA with population data analysis are needed.

The National Health and Nutrition Examination Survey (NHANES) is a systematic survey program conducted by the National Center for Health Statistics to assess the health and nutritional status of children and adults in the United States. It uses a sophisticated multistage probability sampling design to ascertain the prevalence and risk factors for major diseases. An analysis of data from the NHANES database has revealed that accelerated biological aging is associated with OA progression [17]. Therefore, we hypothesize that there is an association between Gastrointestinal diseases and OA and attempt to validate it using NHANES data.

In this study, we analyzed data from the NHANES database to explore the potential association of gastrointestinal diseases with OA development. Subsequently, a logistic regression analysis was performed to further explore the relationship between them. This study might provide valuable insights into OA pathogenesis and inform potential preventive strategies.

## Materials and methods

### Study population

This cross-sectional analysis included 39,156 participants who participated in the NHANES survey between 2011 and 2018. The NHANES is an annual study conducted in the United States. It assesses the health and nutritional status of adults and children by collecting health-related data and information.All the NHANES data are available online (https://wwwn.cdc.gov/nchs/nhanes/search/). All participants provided written informed consent, underwent comprehensive measurements, and participated in standardized interviews. The study protocol was approved by the Ethics Review Board of the National Center for Health Statistics..

### OA definition

The status of the participants regarding having OA was determined using the MCQ160A question “Have you ever been told by a doctor or other health professional that you have arthritis?“and MCQ195 question “What type of arthritis?“. Participants who answered “Yes“to the first question and “OA“to the second question were defined as the OA group. Participants who answered “No” to the first question were categorized in the control group.

### Gastrointestinal diseases definition

Gastrointestinal diseases were defined based on question HSQ510 in the questionnaire section: Was there a gastric or intestinal illness that began with vomiting or diarrhea during the 30-day period? Participants who answered “yes“ were considered to be in the group with gastrointestinal diseases, and those who answered “no” were considered to be in the control group without any gastrointestinal diseases.

### Criteria for participant selection and potential covariates

Based on the OA and gastrointestinal diseases definition, 8,833 participants who participated in the NHANES survey were identified. Participants aged <20 years and those with missing data were excluded. Participants were excluded if their records included missing data or refused to provide data for potential covariates.

These potential covariates included categorical variables: race(non-hispanic whites, non-hispanic blacks, mexican americans and others); gender(male, female); education(below grade 9, grades 9–11, high school graduates, junior college education, education above junior college level); Body Mass Index (BMI) < 24.9 kg/m^2^, 25 kg/m^2^ ≤ BMI ≤ 29.9 kg/m^2^, BMI > 30 kg/m^2^; Alcohol: < 5 cups/day, 5–10 cups/day, > 10 cups/day; smoke:(never smoked or smoked less than 100 cigarettes, smoked at least 100 cigarettes); categorical variables: diabetes (Yes, No), hypertension (Yes, No), cancer (Yes, No), vigorous work activity (Yes, No); continuous variable: age (20–80 years), albumin level (2.6–5.4 g/dL), blood urea nitrogen(BUN) level (0.36–23.21 mmol/L), calcium level (1.9–3.7 mmol/L), creatinine level (26.52–546.31umol/L), phosphorus level(0.581–3.1 mmol/L), uric acid (UA) level(101.1–731.6 umol/L), triglycerides (TG) level (0.203–34.559 mmol/L) and glycohemoglobin level(3.6–16.5%).

### Statistical analysis

The bioinformatics analysis was performed using R software (version 4.2.2; R Foundation for Statistical Computing, Vienna, Austria)‌. Continuous variables were expressed as mean ± standard deviation, while categorical variables were reported as frequency counts with percentages. Three models were designed in this study. Model 1 (unadjusted model) was used to analyze the relationship between gastrointestinal diseases and OA. Model 2 (minimally adjusted model) was adjusted for sex, age, and race. Model 3 (fully adjusted model) was further adjusted for education, alcohol consumption, smoking, BMI, diabetes, hypertension, vigorous work activity, and calcium, creatinine, albumin, BUN, phosphorus, TG, glycated hemoglobin, and UA levels based on model 2.To explore differences in the variables between the OA and control groups, the CreateTableOne function in the TableOne package (v 0.13.2) [18] was used. For categorical variables, the chi-square test, and for continuous variables, the Student’s t-test were applied by default. A baseline table was further constructed (*p* < 0.05). Subsequently, the three models were used to examine the correlation between gastrointestinal diseases and OA and multifactor glm regression models were constructed using the svyglm function from the survey package (v 4.4.2, https://doi.org/10.1214/16-STS605) (*p*<0.05), where all covariates were assumed to interact with OA. To analyze the effects of different covariates and gastrointestinal diseases and OA under different subgroups, stratified analyses were conducted with the covariates in Model 3 (*p < *0.05). A smooth curve was employed to describe the non-linear association between gastrointestinal diseases and OA using the ggplot package (v 3.4.3) [19] based on the adjusted model to further confirm the stability of the association between the two in different populations. After adjusting for covariates, to evaluate the predictive efficiency of gastrointestinal diseases on OA, the pROC package (v 1.18.0) [20] was employed to plot receiver operating characteristic (ROC) curves for model 3 with a threshold of area under the curve (AUC) >0.7.In addition, to detect multicollinearity between BUN and creatinine levels in the model, Spearman correlation analysis was conducted using the “ggpubr” package (V0.6.0, https://CRAN.R-project.org/package=ggpubr) in the NHANES dataset (*p* < 0.05).

## Results

### Multiple covariates exhibited demographic differences between groups

**Fig 1:**
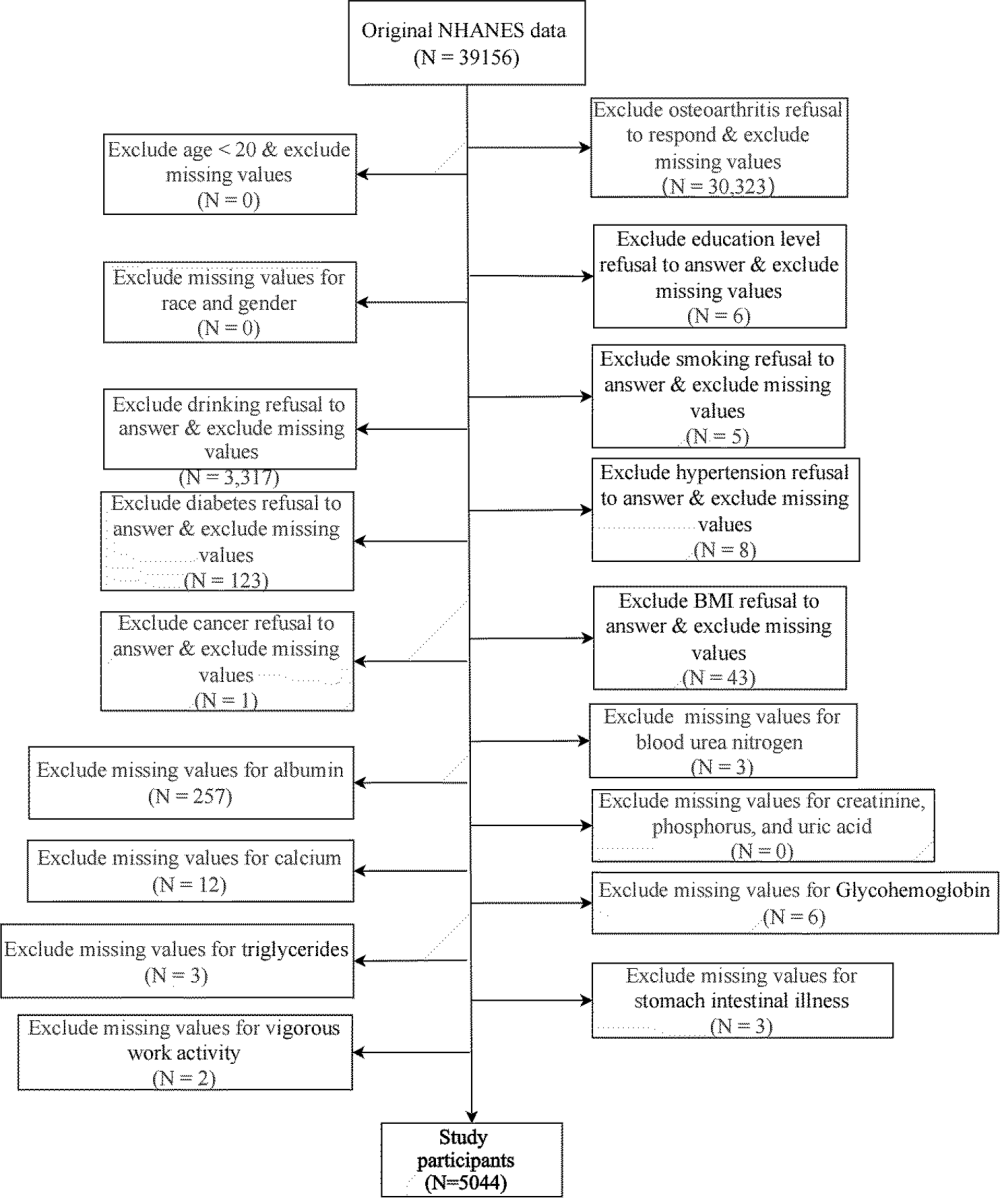
Selection process of the study participants.

Overall, 5,044 participants were recruited for this study Fig 1. describes the inclusion and exclusion process. Participants were categorized into two groups based on their OA status, with 66 having both OA and gastrointestinal diseases, 582 having OA only, 285 having gastrointestinal diseases only, and 4,111 having none. Gastrointestinal diseases had a highly significant impact on OA outcome (*p < *0.001), even after stratification (Table 1). Among the covariates, except for calcium (*p = *0.272), creatinine (*p = *0.056), and UA (*p = *0.475), the remaining clinical characteristics, alcohol consumption (*p < *0.001), albumin (*p < *0.001), BUN (*p < *0.001), phosphorus (*p = *0.007), TG (*p = *0.04), BMI (*p < *0.001), hypertension (*p < *0.001), gender(*p < *0.001), age (*p < *0.001), race (*p < *0.001), education (*p = *0.007), diabetes (*p < *0.001), glycohemoglobin (*p < *0.001), cancer (*p < *0.001), vigorous work activity (*p < *0.001), and smoking (*p < *0.001), all showed significant intergroup differences between the OA and control groups. This indicated that the quality of the included covariates was good. Although calcium, creatinine, and UA did not show significant effects, they were considered common basic adjustment covariates because of their frequent use.

**Table 1:** Baseline statistics of the study population.

		Control	OA	p-value
Variable		(n = 4396)	(n = 648)	
Gastrointestinal Diseases (%)	No	4111 (93.52)	582 (89.81)	<0.001
	Yes	285 (6.48)	66 (10.19)	
Alcohol (%)	Less than 5 Cups/Day	3712 (84.44)	603 (93.06)	<0.001
	5-10 Cups/Day	585 (13.31)	42 (6.48)	
	More than 10 Cups/Day	99 (2.25)	3 (0.46)	
Albumin (mean [SD])		4.25 (0.35)	4.14 (0.34)	<0.001
Blood_urea_nitrogen (mean [SD])		4.64 (1.73)	5.49 (2.16)	<0.001
Calcium (mean [SD])		2.33 (0.09)	2.33 (0.09)	0.272
Creatinine (mean [SD])		76.78 (24.27)	78.77 (27.79)	0.056
Phosphorus (mean [SD])		1.17 (0.18)	1.19 (0.17)	0.007
Uric_acid (mean [SD])		326.38 (82.38)	328.88 (87.96)	0.475
Triglycerides (mean [SD])		1.41 (1.34)	1.52 (1.00)	0.04
BMI (%)	Underweight or Normal	1451 (33.01)	145 (22.38)	<0.001
	Overweight	1486 (33.80)	187 (28.86)	
	Obese	1459 (33.19)	316 (48.76)	
Hypertension (%)	No	3266 (74.29)	280 (43.21)	<0.001
	Yes	1130 (25.71)	368 (56.79)	
Gender (%)	Male	2468 (56.14)	249 (38.43)	<0.001
	Female	1928 (43.86)	399 (61.57)	
Age (mean [SD])		43.21 (16.17)	60.54 (13.37)	<0.001
Race (%)	Mexican American	623 (14.17)	48 (7.41)	<0.001
	Other Race	1188 (27.02)	106 (16.36)	
	Non-Hispanic White	1674 (38.08)	401 (61.88)	
	Non-Hispanic Black	911 (20.72)	93 (14.35)	
Education (%)	Less Than 9th Grade	237 (5.39)	18 (2.78)	0.007
	9-11th Grade	511 (11.62)	72 (11.11)	
	High School Grad	940 (21.38)	132 (20.37)	
	Some College	1388 (31.57)	241 (37.19)	
	College Graduate or above	1320 (30.03)	185 (28.55)	
Diabetes (%)	No	4021 (91.47)	523 (80.71)	<0.001
	Yes	375 (8.53)	125 (19.29)	
Glycohemoglobin (mean [SD])		5.63 (1.04)	5.90 (1.00)	<0.001
Cancer (%)	No	4153 (94.47)	529 (81.64)	<0.001
	Yes	243 (5.53)	119 (18.36)	
Vigorous_work_activity (%)	No	3347 (76.14)	543 (83.80)	<0.001
	Yes	1049 (23.86)	105 (16.20)	
Smoke (%)	non-smoker	2464 (56.05)	279 (43.06)	<0.001
	smoker	1932 (43.95)	369 (56.94)	

### Gastrointestinal diseases as an important exposure factor for OA

**Fig 2:**
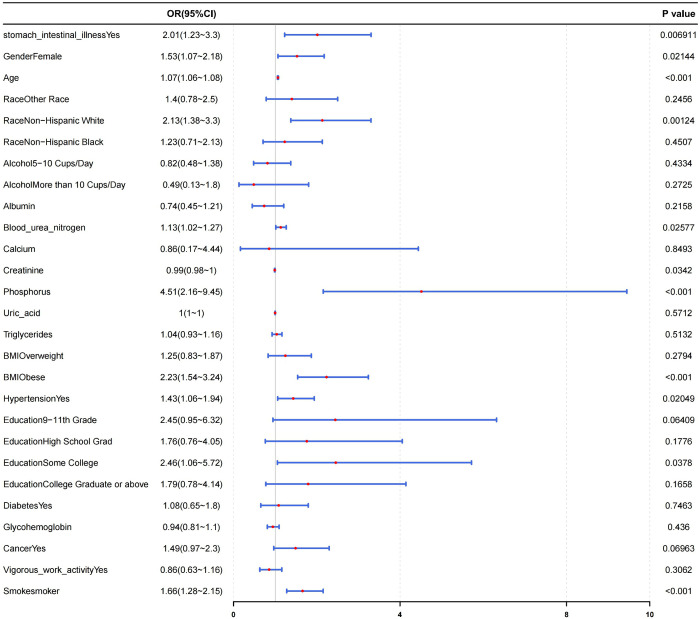
Risk stratification analysis between gastrointestinal diseases (stomach–intestinal illness) and OA risk. The association between stomach–intestinal illness (gastrointestinal diseases) and OA risk was significant (p <0.05). Factors significantly influencing OA development included gender,age,race, blood urea nitrogen level, creatinine level, phosphorus level, body mass index, education, and smoking habit(p <0.05). OA, osteoarthritis.

Based on the results of the above mentioned analyses, the correlation between gastrointestinal diseases and OA was further explored using logistic regression, which demonstrated a strong association (*p* < 0.05), regardless covariates adjustment (Table 2). The OR was 1.71 for model 1 (95% confidence interval [CI]: 1.14–2.57, *p* = 0.0110), 2.27 for model 2 (95% CI: 1.39–3.69, *p* = 0.0014), and 2.01 for model 3 (95% CI: 1.23–3.3, *p* = 0.0069). In all three models, the OR value for gastrointestinal diseases was > 1. Furthermore, all *p*-values for gastrointestinal diseases in the models were <0.05, suggesting that other covariates did not significantly influence the effect of gastrointestinal diseases on OA. Moreover, risk stratification analysis further confirmed the robustness of the association of gastrointestinal diseases with OA in different populations (Fig 2).

**Table 2:** The relationship between exposure factors(Gastrointestinal diseases) and outcome(OA) in the three different models.

Model	OR	95% CI	p-value
model 1	1.71	1.71(1.14-2.57)	0.0110
model 2	2.27	2.27(1.39-3.69)	0.0014
model 3	2.01	2.01(1.23-3.3)	0.0069

OR, odds ratio; CI, confidence interval; OA, osteoarthritis.

### Excellent predictive ability of model 3 For OA

**Fig 3:**
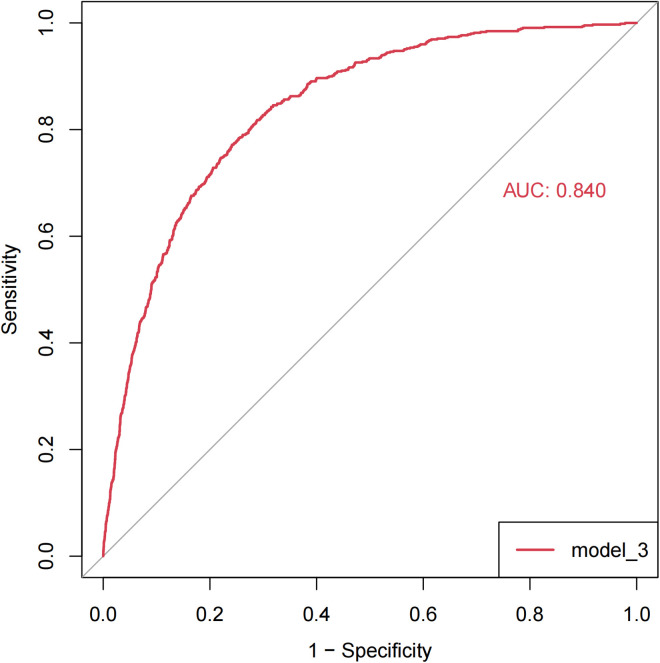
The predictive ability of model 3 for OA. ROC curve for model 3. The ROC curve for model 3 was drawn using the R package “pROC”, and the area under the curve was 0.840. OA, osteoarthritis; ROC, receiver operating characteristic.

**Fig 4:**
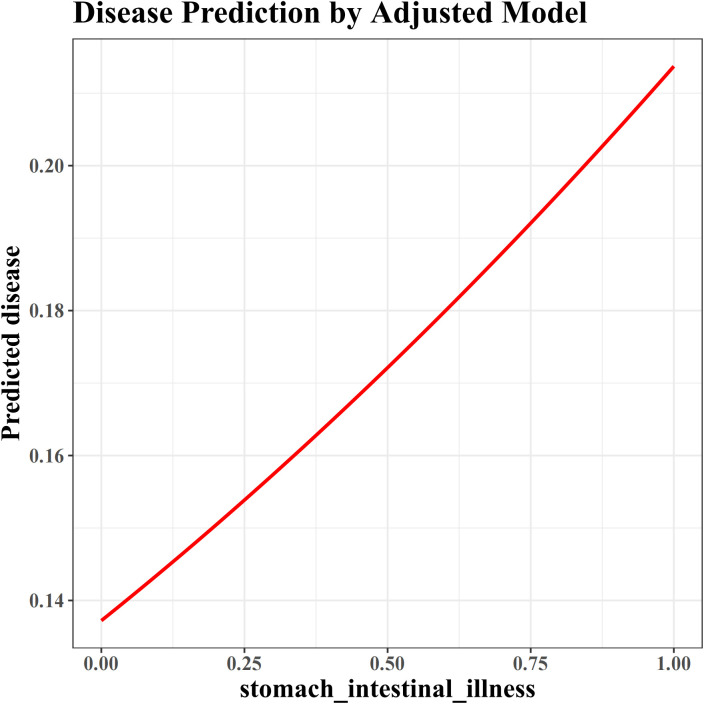
A smooth curve for the association between stomach–intestinal illness (gastrointestinal diseases) and predicted disease(OA). The smooth curve indicated a non-linear association, revealing that the presence of stomach–intestinal illness (gastrointestinal diseases) increased the risk of OA. The horizontal coordinate 0 indicated the absence of stomach–intestinal illness (gastrointestinal diseases) and 1 indicated its presence. OA, osteoarthritis.

An ROC curve was plotted to evaluate the predictive ability of model 3 for OA. Results revealed a relatively accurate predictive effect, as evidenced by an AUC of 0.840 (Fig 3). To confirm the association with OA risk across populations, the model was adjusted using models 2 and 3. The smooth curve depicted a non-linear relationship between gastrointestinal diseases and OA in Fig 4, which revealed that gastrointestinal diseases increased the risk of OA.In addition, the correlation analysis results showed that the correlation between BUN and creatinine levels was not strong (cor = 0.37, *p* < 0.05), indicating no multicollinearity issue (S1 Fig).

## Discussion

This study concluded that gastrointestinal diseases are a significant risk factor for OA. The baseline characteristics of the included participants from the NHANES were analyzed using logistic regression and stratified analyses. The results showed that gastrointestinal diseases significantly impact OA. Furthermore, our analysis suggests that other covariates did not significantly confound the relationship between gastrointestinal diseases and OA. The model’s smooth curve further supports an association between gastrointestinal diseases and OA.

Some observational studies have indicated an association between gastrointestinal diseases and OA. Fernandes et al. focused on a Portuguese cohort consisting of 235 patients with IBD, examining their musculoskeletal manifestations. Approximately 25% of patients with IBD develop extra-intestinal manifestations,which include joint inflammation [11]. Vladimirova et al. conducted a cohort study involving 110 patients with newly diagnosed IBD. Notably, 40% of these patients reported experiencing at least one musculoskeletal symptom, with joint pain and swelling being the most prevalent manifestations [12]. However, the two previous studies predominantly concentrated on the correlation between IBD and musculoskeletal disorders. A notable limitation was their relatively small sample sizes. In contrast, our study has a much larger sample size. Additionally, we have expanded the scope of our research to include all gastrointestinal diseases that present with relevant symptoms. Furthermore, Xu et al. conducted a Mendelian randomization study aimed at assessing the causal relationship between OA and gastrointestinal diseases. They predicted that there is a significant positive correlation between OA and gastroesophageal reflux disease, while no such association with peptic ulcer disease or inflammatory bowel disease [13]. This result is different from the findings of our study. Our study is based on self-reported 30-day disease data, while Xu et al. used genetic instrumental variables in Mendelian randomization. Genetic instrumental variables utilize genetic variations to assess causal relationships, effectively reducing the influence of confounding factors compared to self-reported data. This difference suggests that the relationship between OA and gastrointestinal diseases may vary depending on the research design and data type, necessitating further exploration and validation.

The baseline statistics show that the OA group has a higher incidence of cancer and diabetes. This may be linked to metabolic syndrome in patients with OA, characterized by obesity and insulin resistance, which can increase diabetes risk by affecting insulin secretion and action [21]. Moreover, OA, diabetes, and cancer may share common risk factors such as age, obesity, and lack of exercise. These factors can simultaneously influence multiple disease occurrences, leading to higher rates of diabetes and cancer among patients with OA. Additionally, these shared risk factors might also impact gastrointestinal disease development, further influencing the relationship between gastrointestinal issues and OA.

Despite our promising findings, the mechanism by which gastrointestinal diseases increase the risk of OA remains unclear. The chronic inflammatory state and dysbiosis of the flora may explain the association.Although the pathogenesis of OA has not been fully elucidated, existing evidence suggests that inflammation and immune disorders jointly contribute to structural damage of the cartilage [2,4]. Chronic inflammation of the gastrointestinal tract, such as that in IBD, is not limited to the gastrointestinal tract. It can also affect other body parts, including bones and joints, through various complex mechanisms, leading to arthritis, osteoporosis, and other diseases [1,11 2]. Some research has indicated that patients with IBD are at a higher risk of developing OA [1 2]. Furthermore, gastrointestinal diseases such as tumors and peptic ulcers can alter the composition and function of the gut microbiota, disrupting the microbial balance within the gastrointestinal tract [22,23]. Lipopolysaccharide, a constituent of the outer layer of the cell wall in gram-negative bacteria, is regarded as a primary factor triggering a mild systemic inflammatory response [14,16]. An imbalance in the intestinal flora can lead to intestinal mucosal leakage and increase the blood level of lipopolysaccharide, resulting in a mild systemic inflammatory response and immune system disorder. Additionally, these patients may also have other chronic diseases, such as metabolic syndrome [21,24]. These diseases further exacerbate OA progression through various mechanisms, including metabolic disorders and oxidative stress [24]. Consequently, a long-term, chronic inflammatory state increases the likelihood of joint inflammation and injury. However, the role of these mechanisms remains speculative and requires further research to verify their specific impact on OA.

This study revealed considerable intergroup differences in variables such as alcohol consumption, albumin and TG levels, BMI, hypertension, age, and glycated hemoglobin level between the OA and control groups. Shen et al. found that a high BMI is independently associated with an increased risk of OA through a two-sample Mendelian randomization analysis [25]. He et al. analyzed data from the NHANES database from 2005–2010–2015–2018, involving 50,259 participants, and the results showed that alcohol consumption plays an important role in promoting the development of OA [26]. Huang et al. analyzed data from the NHANES database to examine the potential association between the TG index and the increased risk of OA. They found that TG may be a valuable predictor of OA and provided a new perspective for evaluating and treating OA [27]. Tsukada et al. suggested that glycosylated hemoglobin is a potential risk factor for knee OA [28]. Our results are consistent with these findings. Given that OA is affected by multiple risk factors, avoiding and managing these risk factors is of significance for OA prevention and treatment.

The findings of this study may offer a framework for evidence-based interventions and have implications for clinical practice and public health policy. Clinically, recognizing gastrointestinal disorders as OA risk factors enables healthcare professionals to adopt proactive prevention strategies for affected patients. From a public health perspective, strategic resource allocation and augmented funding for gastrointestinal diseases and OA disease management programs are essential. Furthermore, systematic implementation of early screening protocols would enable prompt diagnosis and intervention, ultimately alleviating the societal burden of these conditions.

Notwithstanding these contributions, several limitations must be considered. The external validity of our conclusions is circumscribed by the demographic composition of our cohort, which was predominantly derived from American populations. Methodologically, as our study was based on a bioinformatics analysis, complementary in vitro and in vivo validation is required to delineate the precise molecular cascades linking gastrointestinal dysfunction to chondrocyte degradation pathways.Lastly, self-reported data may misclassify gastrointestinal disease status due to memory bias or subjective judgment, failing to accurately reflect true health conditions. Furthermore, the constraints associated with sample size and the selection bias inherent in secondary data analysis may adversely affect both the statistical power and the generalizability of the research findings. Therefore, future research should employ more precise disease measurement methods and enhance control over confounding factors to improve reliability. Additionally, increasing the sample size and including relevant clinical diagnostic information will allow for a better analysis of the association between gastrointestinal diseases and study outcomes, providing a solid theoretical foundation for clinical interventions.

## Conclusions

By analyzing observational data from the NHANES, our study provides strong evidence that gastrointestinal diseases contribute to OA development. This finding provides valuable insights into OA pathogenesis and informs potential preventive strategies.

## Supporting information

S1 Fig Correlation analysis between blood urea nitrogen and creatinine. (TIF)
