# Supplementary figures and images for: Association between gastrointestinal diseases and osteoarthritis risk based on data from NHANES 2011–2018
Source: PLoS One. 2025 Aug 13;20(8):e0330064. doi: 10.1371/journal.pone.0330064 (PMC12349171; doi:10.1371/journal.pone.0330064)

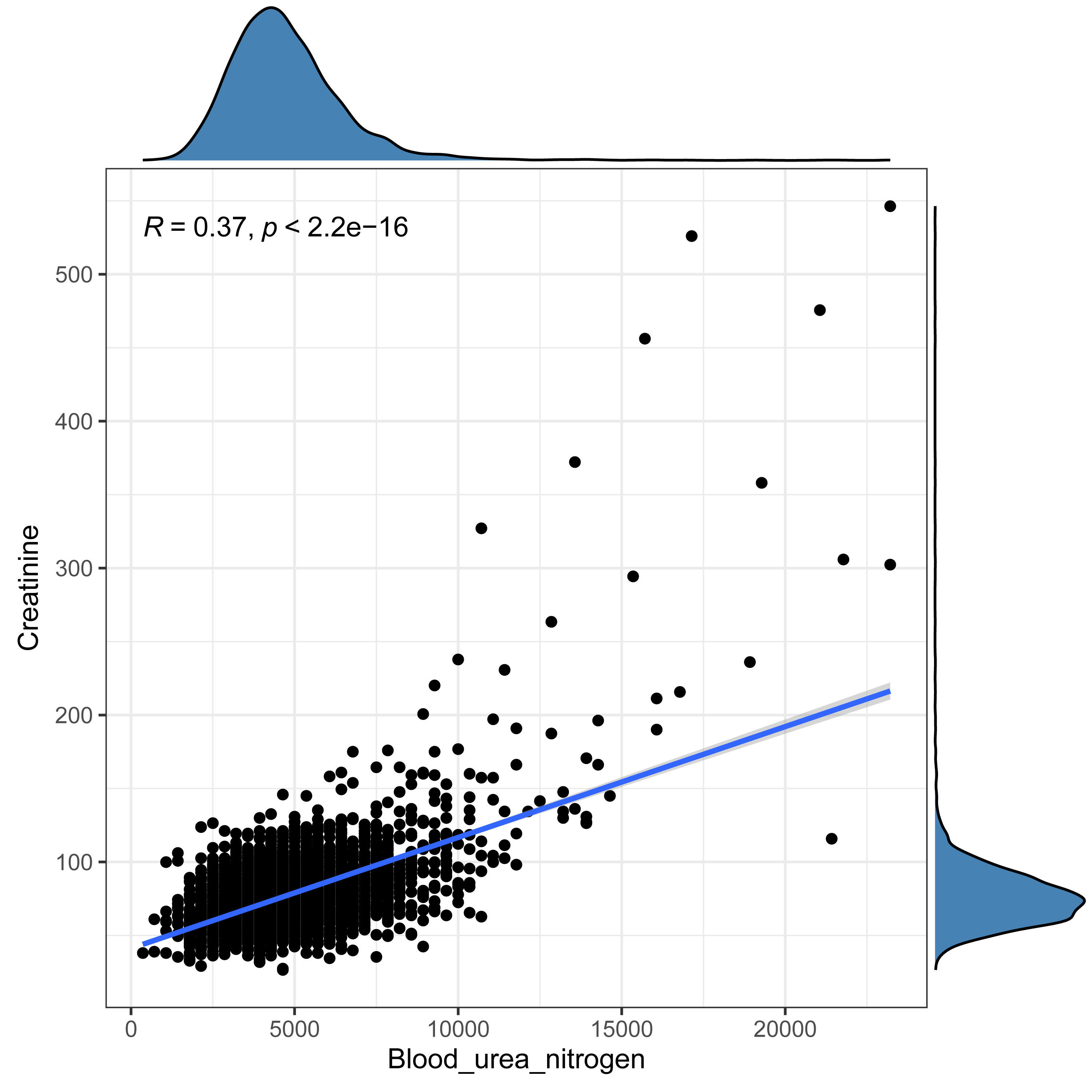

Supplement: S1 Fig — (TIF) [file pone.0330064.s001.tif]
